# Perioperative anesthetic management in patients undergoing resection of giant abdominal masses: a retrospective analysis of 21 cases

**DOI:** 10.3389/fonc.2026.1791854

**Published:** 2026-04-23

**Authors:** Xiaoxia Zhang, Xiaoli Sun, Miao He, Han Wei, Mingying Li

**Affiliations:** Department of Anesthesiology, Beijing Chao-Yang Hospital, Capital Medical University, Beijing, China

**Keywords:** abdominal mass, anesthetic management, case series, giant tumor, perioperative management

## Abstract

**Objective:**

To summarize perioperative anesthetic management in patients undergoing resection of giant abdominal masses.

**Methods:**

Perioperative data from 21 patients who underwent resection of giant abdominal masses between January 2016 and December 2024 were retrospectively analyzed. Descriptive statistical methods and stratified comparative analyses were applied to evaluate perioperative characteristics and outcomes.

**Results:**

The maximum tumor diameter ranged from 15 to 50 cm. Tumors originated from the abdominal wall in 8 cases (38.10%), the abdominopelvic cavity in 6 cases (28.57%), and the retroperitoneum in 7 cases (33.33%). Pathological diagnoses predominantly included malignant tumors in 11 cases (52.38%) and borderline tumors in 7 cases (33.33%). General anesthesia was administered to all 21 patients, with single-lumen endotracheal intubation used in 16 cases (76.2%). Intraoperative monitoring consisted of invasive arterial pressure monitoring in 16 cases (76.2%), central venous pressure monitoring in 15 cases (71.4%), and FloTrac-based cardiac output monitoring in 8 cases (38.1%). Operative duration ranged from 110 to 440 min, and massive hemorrhage (≥ 1,000 mL) occurred in 57.1% of patients. Postoperative intensive care unit (ICU) stay ranged from 0 to 12 days, while postoperative hospital stay ranged from 7 to 58 days. Stratified analyses indicated that patients with retroperitoneal masses presented with higher preoperative American Society of Anesthesiologists (ASA) physical status classification, larger tumor diameter, and longer ICU and hospital stays. Patients with malignant tumors were older and demonstrated higher ASA classification, longer operative duration, and prolonged ICU stay.

**Conclusion:**

Anesthetic management for resection of giant abdominal masses requires individualized strategies. Preoperative multidisciplinary consultation and interventional embolization may reduce surgical risk. Intraoperative advanced hemodynamic monitoring and meticulous volume management warrant particular attention.

## 1 Introduction

Giant abdominal masses are defined as neoplastic lesions arising within the abdominal region, including the abdominal wall, intra-abdominal cavity, and retroperitoneum, with a diameter exceeding 15 cm or a weight greater than 2 kg (1). Although these lesions are relatively uncommon, their substantial volume can exert pronounced effects on the respiratory and circulatory systems and disrupt internal homeostasis, thereby creating considerable challenges for surgical intervention and perioperative anesthetic management (2). The cardiopulmonary effects associated with giant abdominal masses vary according to anatomical location, and differences in pathological characteristics further influence perioperative risk profiles and management approaches.

Currently, the published literature on anesthetic management for resection of giant abdominal masses consists predominantly of case reports, with limited systematic analyses comparing anesthetic strategies across different anatomical locations and pathological types. The present retrospective analysis evaluated perioperative anesthetic management data from 21 patients who underwent resection of giant abdominal masses. The objective was to summarize anesthetic management characteristics associated with different anatomical sites and pathological classifications, identify key perioperative risk factors, and provide evidence to support stratified anesthetic management strategies in clinical practice.

## 2 Materials and methods

### 2.1 Study design

A single-center retrospective case series design was used for this study. The study protocol was approved by the institutional Medical Ethics Committee (approval No. 2025-Sci-1037). All procedures were conducted in accordance with the ethical principles of the Declaration of Helsinki. Patient data were anonymized prior to analysis.

### 2.2 Study population

Eligibility criteria included age 18 to 80 years, presence of an abdominal mass involving the abdominal wall, intra-abdominal cavity, or retroperitoneum with a maximum diameter of at least 15 cm or a weight of at least 2 kg, planned surgical resection of a giant abdominal mass, and availability of complete perioperative data. Exclusion criteria included emergency surgery, American Society of Anesthesiologists (ASA) physical status classification ≥ IV, pregnancy, and incomplete clinical records. During the study period, 24 patients met the diagnostic criteria for a giant abdominal mass. Three patients were excluded because of incomplete data, and 21 patients were included in the final analysis.

### 2.3 Data collection

Data were collected from the hospital electronic medical record system, anesthesia information system, operative record system, and nursing documentation system. Collected variables included demographic characteristics, mass-related features, preoperative preparation, anesthetic management, intraoperative management, and perioperative outcomes.

### 2.4 Statistical analysis

Due to sample size limitations, in addition to reporting p-values, this study calculated the effect size to assess the actual magnitude of clinical differences. For the comparison between the two groups, r=Z/√N is used as the effect size indicator (Z is the Z-value of Mann Whitney U test, N is the total sample size). Explanation criteria for effect size: small effect (0.1-0.3), moderate effect (0.3-0.5), large effect (>0.5).

The distribution of continuous variables was evaluated using the Shapiro–Wilk test in conjunction with histogram inspection and normal Q–Q plots. Continuous variables with a normal distribution were presented as mean ± standard deviation, whereas non-normally distributed variables were summarized as median with interquartile range. Owing to the limited overall sample size (*N* = 21) and small subgroup sizes, nonparametric methods were applied for all between-group comparisons of continuous variables to minimize potential bias related to distributional assumptions. The Mann–Whitney U test was used for comparisons between two groups, and the Kruskal–Wallis H test was applied for comparisons among multiple groups. Categorical variables were expressed as frequencies and percentages, and between-group comparisons were performed using the Fisher’s exact test.

Statistical analyses were conducted using SPSS version 27.0, and a two-sided *p* value < 0.05 was considered statistically significant.

## 3 Results

Normality of continuous variables was assessed using the Shapiro–Wilk test in conjunction with normal Q–Q plots. Preoperative general variables, including age, height, body weight, and body mass index (BMI), followed a normal distribution and were therefore summarized as mean ± standard deviation. Most perioperative management and surgery-related variables did not conform to a normal distribution and were consequently reported as median with interquartile range to ensure consistency across tables.

### 3.1 Baseline characteristics

The study cohort comprised 6 male patients (28.57%) and 15 female patients (71.43%). Age ranged from 24 to 79 years, with a mean of 50.29 ± 17.95 years. BMI ranged from 16.4 to 31.9, with a mean of 24.49 ± 4.27.

#### 3.1.1 ASA classification

One patient was classified as ASA physical status I (4.76%), 10 patients as ASA II (47.62%), and 10 patients as ASA III (47.62%). Major comorbidities included hypertension in 10 patients (47.61%), coronary artery disease in 3 patients (14.28%), diabetes mellitus in 3 patients (14.28%), anemia in 10 patients (47.61%), hypoproteinemia in 10 patients (47.61%), coagulation abnormalities in 6 patients (28.57%), and hypoxemia in 4 patients (19.05%).

#### 3.1.2 Pulmonary function

Pulmonary function testing was not completed in 2 patients (9.52%) because of noncompliance. Severe restrictive ventilatory dysfunction was identified in 1 patient (4.76%), moderate restrictive ventilatory dysfunction in 2 patients (9.52%), moderate mixed ventilatory dysfunction in 2 patients (9.52%), and mild restrictive ventilatory dysfunction in 1 patient (4.76%).

#### 3.1.3 Cardiac function

Cardiac insufficiency was present in 1 patient (4.8%), and borderline reduced left ventricular ejection fraction was observed in 2 patients (9.5%).

### 3.2 Mass characteristics

#### 3.2.1 Origin of masses

Masses originated from the abdominal wall in 8 patients (38.10%), the abdominopelvic cavity in 6 patients (28.57%), and the retroperitoneum in 7 patients (33.33%).

#### 3.2.2 Extent of tumor involvement

Tumor involvement was confined to the abdominal wall in 8 patients (38.10%), the abdominopelvic cavity in 3 patients (14.29%), and the retroperitoneum in 2 patients (9.52%). Combined abdominopelvic and retroperitoneal involvement was observed in 8 patients (38.10%).

#### 3.2.3 Mass dimensions

**Figure 1:**
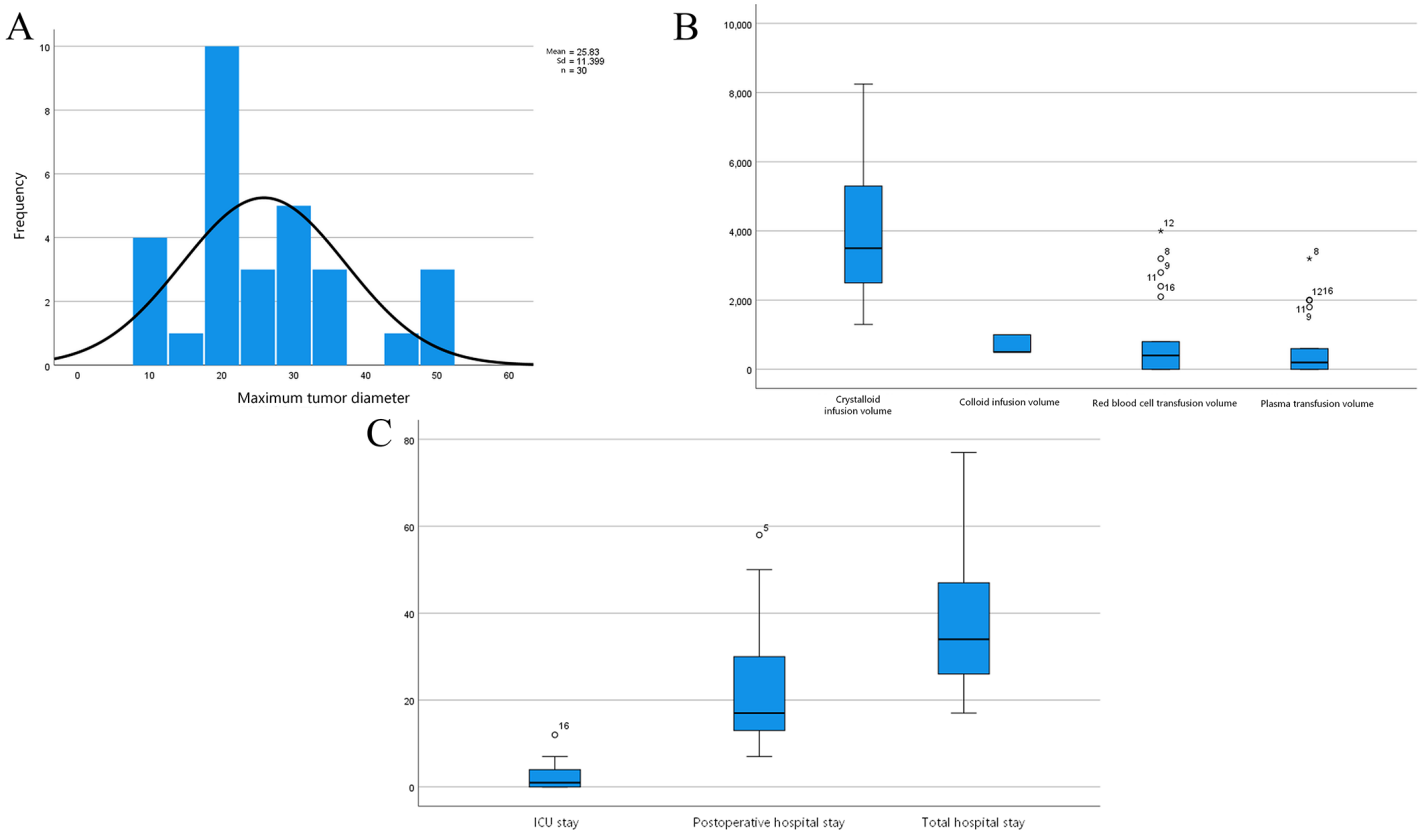
Overall perioperative characteristics of patients undergoing resection of giant abdominal masses. **(A)** Distribution of maximum tumor diameter; **(B)** Box plots of intraoperative fluid administration and blood product transfusion volumes, including crystalloid infusion volume, colloid infusion volume, red blood cell transfusion volume, and plasma transfusion volume; **(C)** Box plots of perioperative duration variables, including intensive care unit stay, postoperative hospital stay, and total hospital stay.

The number of resected tumors per patient ranged from 1 to more than 200. Maximum tumor diameter ranged from 10 to 50 cm, with a median of 23.75 (19.75, 30.75) cm. The distribution of tumor diameter is presented in **Figure 1**.

#### 3.2.4 Pathological classification

Malignant tumors were identified in 11 patients (52.38%), including liposarcoma (4 patients), fibrosarcoma (1 patient), leiomyosarcoma (3 patients), high-risk gastrointestinal stromal tumor (1 patient), adult-type ovarian granulosa cell tumor (1 patient), and malignant spindle cell tumor (1 patient). Borderline tumors were identified in 7 patients (33.33%), comprising desmoid-type fibromatosis (4 patients), kaposiform hemangioendothelioma (1 patient), myofibroblastoma (1 patient), and sclerosing stromal tumor (1 patient). Benign tumors were present in 3 patients (14.29%), including neurofibroma in 2 patients and intravenous leiomyomatosis in 1 patient.

One patient with histopathologically benign intravenous leiomyomatosis was categorized within the malignant tumor group for stratified analysis. This classification is supported by the following evidence-based rationale:

First, although histologically benign, intravenous leiomyomatosis (IVL) exhibits biological behavior characteristic of malignant tumors, including intravascular invasion, metastatic spread, and potential extension to the right heart and pulmonary artery (3, 4). As described in large cohort studies by Chen et al. (5), IVL is defined as a histologically-benign tumor with biological behavior similar to that of a malignant tumor.

Second, the perioperative risk profile of IVL aligns with malignant tumors rather than benign lesions. Stage III-IV IVL carries a documented risk of sudden death due to cardiac involvement or pulmonary embolism (5), and surgical management requires multidisciplinary collaboration involving anesthesiology, cardiovascular surgery, and gynecology, whose complexity comparable to malignant retroperitoneal tumors (6).

Third, previous clinical studies analyzing perioperative outcomes in IVL have consistently employed risk stratification paradigms that prioritize biological behavior over histopathological diagnosis, classifying these cases within high-risk groups for surgical planning and prognostic evaluation (6, 7).

Therefore, to ensure clinical relevance of our stratified analysis, this case was retained in the malignant tumor group. Reclassification into the benign group would misrepresent the actual perioperative risk and contradict established clinical management protocols.

### 3.3 Preoperative preparation

Multidisciplinary team consultation was conducted in 11 patients (52.4%). Preoperative vascular interventional procedures were performed in 13 patients (57.1%). One patient underwent tumor angiography without indication for embolization, whereas the remaining 12 patients received tumor vascular embolization. Among these, 3 patients underwent embolization on two or more occasions. In one patient, two sessions of tumor arterial embolization were performed during preoperative evaluation, followed by placement of aortic and iliac artery occlusion balloons before tumor resection to facilitate intraoperative blood flow control and reduce blood loss. Double-J ureteral stents were placed in 7 patients (33.3%).

### 3.4 Anesthetic management

#### 3.4.1 Anesthesia technique

General anesthesia was administered to all patients. A laryngeal mask airway was used in 4 patients (19.05%), single-lumen endotracheal intubation in 16 patients (76.19%), and double-lumen endotracheal intubation in 1 patient (4.76%). Bilateral transversus abdominis plane block was applied as adjunctive analgesia in 6 patients (28.57%). Combined neuraxial anesthesia was not used in any patient. The median number of intravenous access sites was two.

#### 3.4.2 Intraoperative monitoring

Invasive arterial pressure monitoring was applied in 16 patients (76.2%), central venous pressure monitoring in 15 patients (71.4%), and FloTrac-based cardiac output monitoring in 8 patients (38.0%). The median number of intraoperative arterial blood gas analyses was three.

### 3.5 Intraoperative fluid and blood product management

Crystalloid infusion volume ranged from 1,300 to 8,250 mL, with a median of 3,500 (2,300, 5,650) mL. Colloid infusion volume ranged from 500 to 1,000 mL, with a median of 500 (500, 1,000) mL. Red blood cell transfusion was administered in 12 patients (57.1%), with volumes ranging from 0 to 4,000 mL and a median of 800 (0, 1,450) mL. Plasma transfusion was administered in 10 patients (47.6%), with volumes ranging from 0 to 3,200 mL and a median of 200 (0, 1,200) mL. Box plots of these variables are presented in **Figure 1B**. Vasoactive agents were required in 14 patients (66.7%), including norepinephrine in 13 patients (61.9%), dopamine in 5 patients (23.8%), and ephedrine in 4 patients (19.0%).

### 3.6 Surgical and perioperative outcomes

Surgical duration ranged from 110 to 440 min, with a median of 185 (150, 282) min. Blood loss ranged from 200 to 7,500 mL, with a median of 1000 (350, 3,000) mL. Massive hemorrhage (≥ 1,000 mL) occurred in 12 patients (57.1%). Immediate postoperative extubation was achieved in 13 patients (61.9%), whereas 8 patients (38.1%) remained intubated. Patient-controlled intravenous analgesia was applied in 16 patients (76.2%). Ten patients (47.6%) required admission to the intensive care unit (ICU). ICU stay ranged from 0 to 12 days, with a median of 1 (0, 4.5) day. Postoperative hospital stay ranged from 7 to 58 days, with a median of 17.0 (11.5, 31.0) days, and total hospital stay ranged from 17 to 67 days, with a median of 34.0 (25.5, 47.0) days. Box plots of ICU stay, postoperative hospital stay, and total hospital stay are presented in **Figure 1C**.

### 3.7 Perioperative complications

The overall complication rate was 52.6% (11 patients). Major complications included surgical site infection or subcutaneous fluid collection in 6 patients (28.6%), bilateral lower-lobe atelectasis in 2 patients (9.5%), lower-extremity deep vein thrombosis in 2 patients (9.5%), postoperative acute myocardial injury with cardiac dysfunction in 1 patient (4.8%), hemorrhagic shock in 1 patient (4.8%), postoperative sepsis in 1 patient (4.8%), intra-abdominal infection in 1 patient (4.8%), and pancreatitis or pancreatic fistula in 1 patient (4.8%). No perioperative mortality was observed.

### 3.8 Stratified analysis

#### 3.8.1 Stratification by mass origin

Patients were stratified into abdominal wall, abdominopelvic, and retroperitoneal groups. No significant differences were identified among groups with respect to age or BMI. Statistically significant differences were observed in sex distribution (*p* = 0.027), ASA classification (*p* = 0.049), and maximum tumor diameter (*p* = 0.016). Pairwise comparisons demonstrated a significant difference in sex distribution between the abdominopelvic and abdominal wall groups (*p* = 0.020), with a higher proportion of female patients in the abdominopelvic and retroperitoneal groups. ASA classification differed significantly between the retroperitoneal and abdominal wall groups (*p* = 0.022), with higher classification in the retroperitoneal group. Maximum tumor diameter was significantly larger in the retroperitoneal group compared with the abdominal wall group (*p* = 0.009) and the abdominopelvic group (*p* = 0.030) (**Table 1**).

**Table 1:** Comparison of preoperative general characteristics stratified by mass origin.

No.	Group	Age (years)	Sex (male/female), n	BMI	ASA	Maximum tumor diameter (cm)
1	Abdominal wall	47.75±21.38	5/3	25.55±3.55	2.13±0.64	23.38±9.97
2	Abdominopelvic	53.00±16.20	0/6	22.67±4.82	2.33±0.52	25.00±6.07
3	Retroperitoneal	50.86±18.47	1/6	24.83±4.39	2.85±0.38	39.86±11.92

Preoperative preparation measures included multidisciplinary team consultation, tumor vascular embolization, and ureteral stent placement. Significant differences among groups were identified for multidisciplinary team consultation (*p* = 0.016) and ureteral stent placement (*p* = 0.011). Pairwise analysis indicated that multidisciplinary team consultation was more frequently performed in the abdominopelvic and retroperitoneal groups than in the abdominal wall group (*p* = 0.044 and *p* = 0.006, respectively), with no difference between the abdominopelvic and retroperitoneal groups (*p* = 0.435). Ureteral stent placement was more frequent in the abdominopelvic group than in the abdominal wall and retroperitoneal groups (*p* = 0.011 and *p* = 0.017, respectively), whereas no difference was observed between the latter two groups (*p* = 0.063) (**Table 2**).

**Table 2:** Comparison of preoperative preparation measures stratified by mass origin.

No.	Group	MDT consultation (Yes/No)	Vascular embolization (Yes/No)	Ureteral stent placement (Yes/No)
1	Abdominal wall	1/7	4/4	1/7
2	Abdominopelvic	4/2	2/4	5/1
3	Retroperitoneal	6/1	6/1	1/6

Analysis of anesthetic management demonstrated no significant differences among groups in the use of invasive arterial pressure monitoring (*p* = 0.181), central venous pressure monitoring (*p* = 0.094), peripheral arterial cardiac output monitoring (*p* = 0.175), adjunctive regional analgesia (*p* = 0.082), or postoperative intravenous analgesia (*p* = 0.099) (**Table 3**).

**Table 3:** Comparison of anesthetic management characteristics stratified by mass origin.

No.	Group	Invasive arterial pressure (Yes/No)	Central venous pressure (Yes/No)	FloTrac monitoring (Yes/No)	Adjunctive regional analgesia (Yes/No)	Postoperative intravenous analgesia (Yes/No)
1	Abdominal wall	6/2	3/5	1/7	0/8	8/0
2	Abdominopelvic	6/0	5/1	3/3	3/3	3/3
3	Retroperitoneal	7/0	6/1	4/3	3/4	5/2

**Figure 2:**
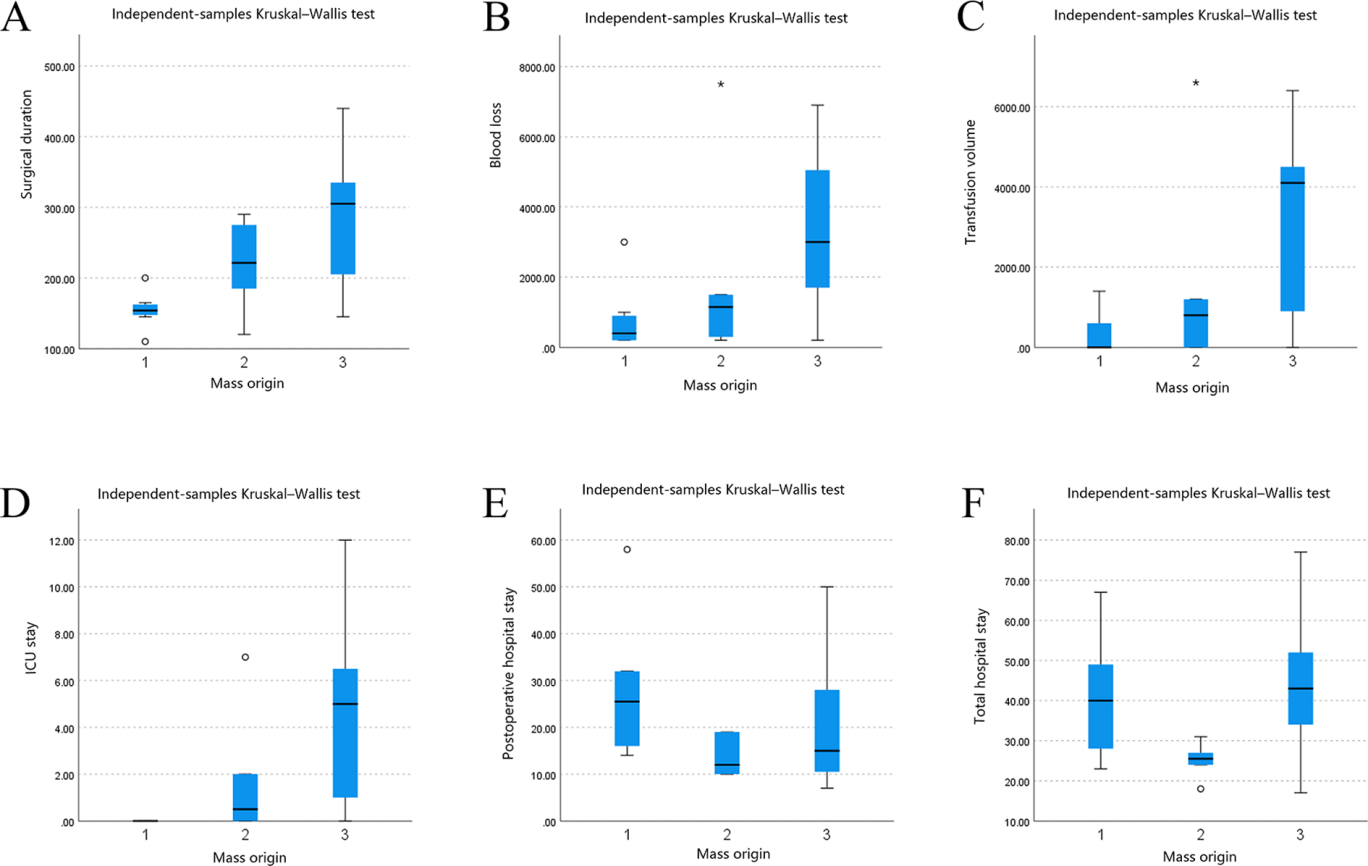
Comparison of surgery-related variables by mass origin. 1 Abdominal wall; 2 Abdominopelvic 3 Retroperitoneal.

Surgery-related variables demonstrated significant differences among groups for surgical duration (*p* = 0.026), ICU stay (*p* = 0.006), and total hospital stay (*p* = 0.026), with longer durations observed in the retroperitoneal group. Overall comparisons for blood loss (*p* = 0.083), transfusion volume (*p* = 0.067), and postoperative hospital stay (*p* = 0.111) were not statistically significant. *Post hoc* pairwise comparisons indicated that patients in the retroperitoneal group had longer surgical duration (Z = 2.377, *p* = 0.017, r = 0.614, 95% CI [0.857,0.148]), greater blood loss (Z = 2.166, *p* = 0.030, r = 0.559, 95% CI [0.833,0.066]), higher transfusion volume (Z = 2.318, *p* = 0.020, r = 0.599, 95% CI [0.850,0.124]), and longer ICU stay (Z = 3.137, *p* = 0.002, r = 0.810, 95% CI [0.934, 0.509]) than those in the abdominal wall group. Given the small sample size, these findings should be interpreted as exploratory and hypothesis-generating rather than definitive. The observed large effect sizes suggest clinically meaningful differences that warrant validation in larger cohorts. As the overall tests did not reach statistical significance, these findings should be interpreted cautiously and require confirmation in larger cohorts (**Table 4** and **Figure 2**).

**Table 4:** Comparison of surgery-related variables stratified by mass origin.

No.	Group	Surgical duration (min)	Blood loss (mL)	Transfusion volume (mL)	ICU stay (days)	Postoperative hospital stay (days)	Total hospital stay (days)
1	Abdominal wall	154 (146,164)	400 (200,950)	0 (0,0)	0(0,0)	25.5(16.0,32.0)	40.0(28.0,49.0)
2	Abdominopelvic	222 (169,279)	1150 (275,3000)	800 (0,1200)	0.5(0,2)	12.0(10.0,19.0)	25.5(24.0,27.0)
3	Retroperitoneal	305 (180,360)	3000 (1000,5600)	4100 (900,4500)	5.0(1,6.5)	15.0(10.5,28.0)	43.0(34.0,52.0)

#### 3.8.2 Stratification by pathological nature

Patients were further stratified into a malignant tumor group and a non-malignant tumor group, the latter including borderline and benign tumors. Analysis of preoperative characteristics demonstrated that age (*p* = 0.023) and ASA classification (*p* = 0.037) were significantly higher in the malignant tumor group. Although the mean maximum tumor diameter was greater in the malignant tumor group, the difference was not statistically significant (*p* = 0.251) (**Table 5**).

**Table 5:** Comparison of preoperative general characteristics stratified by pathological nature.

No.	Group	Age (years)	Sex (male/female), n	BMI	ASA	Maximum tumor diameter (cm)
1	Malignant	57.17±13.41	3/9	25.49±4.02	2.67±0.49	31.42±11.60
2	Non-malignant	41.11±19.80	3/6	23.16±4.47	2.11±0.60	26.56±12.77

No significant differences were observed between groups in preoperative multidisciplinary team consultation (*p* = 0.198), tumor vascular embolization (*p* = 1.000), or ureteral stent placement (*p* = 0.642) (**Table 6**).

**Table 6:** Comparison of preoperative preparation measures stratified by pathological nature.

No.	Group	MDT consultation (Yes/No)	Vascular embolization (Yes/No)	Ureteral stent placement (Yes/No)
1	Malignant	8/4	7/5	5/7
2	Non-malignant	3/6	5/4	2/7

Anesthetic management did not differ significantly between groups with respect to invasive arterial pressure monitoring (*p* = 0.171), central venous pressure monitoring (*p* = 0.159), peripheral arterial cardiac output monitoring (*p* = 0.367), or postoperative intravenous analgesia (*p* = 0.338). Adjunctive regional analgesia was applied more frequently in the malignant tumor group, and this difference was statistically significant (*p* = 0.019) (**Table 7**).

**Table 7:** Comparison of anesthetic management characteristics stratified by pathological nature.

No.	Group	Invasive arterial pressure (Yes/No)	Central venous pressure (Yes/No)	FloTrac monitoring (Yes/No)	Adjunctive regional analgesia (Yes/No)	Postoperative intravenous analgesia (Yes/No)
1	Malignant	12/0	10/2	6/6	6/6	8/4
2	Non-malignant	7/2	4/5	2/7	0/9	8/1

**Figure 3:**
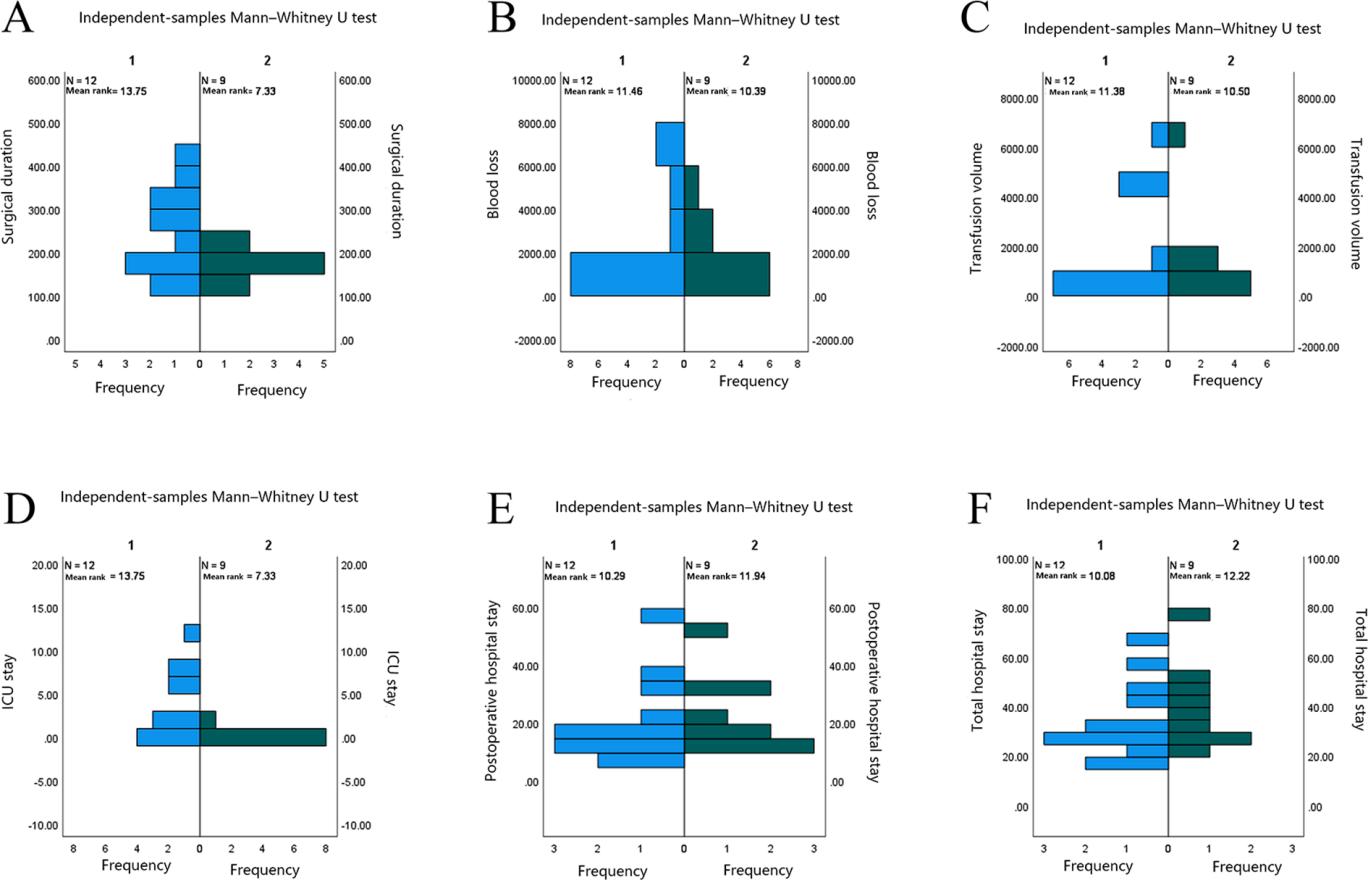
Comparison of surgery-related variables by pathological nature. 1 Malignant tumors; 2 Non-malignant tumors (borderline and benign).

Surgical outcomes differed significantly between groups for surgical duration (*p* = 0.018) and ICU stay (*p* = 0.018), both of which were longer in the malignant tumor group. Although median blood loss and transfusion volume were higher in the malignant tumor group, these differences did not reach statistical significance (*p* = 0.702 and *p* = 0.754, respectively) (**Table 8** and **Figure 3**).

**Table 8:** Comparison of surgery-related variables stratified by pathological nature.

No.	Group	Surgical duration (min)	Blood loss (mL)	Transfusion volume (mL)	ICU stay (days)	Postoperative hospital stay (days)	Total hospital stay (days)
1	Malignant	260 (175,308)	1050 (250,4300)	800 (0,4250)	1.5(0.0,6.5)	17(10,25.5)	29.5(25,45)
2	Non-malignant	158 (150,180)	800 (400,2400)	800 (0,1200)	0(0,0)	17(14,32)	39(28,45)

## 4 Discussion

### 4.1 Clinical implications of patient characteristics and mass features

The study population primarily comprised middle-aged and older patients, with a predominance of women, and most patients were classified as ASA II or III. These characteristics are consistent with previously reported epidemiological profiles of giant abdominal masses (8). Notably, abnormal pulmonary function was present in 47.62% of patients. Among these, two patients were unable to cooperate with pulmonary function testing, and one patient demonstrated severe restrictive ventilatory dysfunction, indicating that giant abdominal masses exert substantial adverse effects on respiratory function (2).

From a pathophysiological standpoint, the systemic effects of giant abdominal masses are largely attributable to mechanical compression, alterations in thoracoabdominal pressure, and increased metabolic demand (1). Compression of the inferior vena cava can directly impair venous return, resulting in reduced preload and diminished cardiac filling, particularly affecting right ventricular function (9). Compensatory neurohumoral activation induces peripheral vasoconstriction, leading to increased afterload. Sustained exposure to this altered hemodynamic state predisposes patients to cardiac dysfunction. In the present cohort, 14.29% of patients exhibited cardiac dysfunction or reduced left ventricular systolic function, which is consistent with these mechanisms.

In addition, relatively high proportions of patients presented with anemia (47.6%), hypoproteinemia (28.6%), and coagulation abnormalities (28.6%). These findings reflect the hypermetabolic and consumptive effects associated with giant abdominal masses and indicate the presence of chronic inflammation and disordered nutritional metabolism (10). The coexistence of multiple physiological derangements further reduced tolerance to surgical intervention and anesthesia.

Most masses in this cohort were malignant or borderline in nature and exhibited extreme size, with a maximum diameter of 50 cm and a maximum weight of 13 kg. Such dimensions resulted in marked compression of adjacent organs and major vascular structures.

The classification of intravenous leiomyomatosis within the malignant tumor group requires further elaboration. While histopathologically benign, this entity demonstrates several malignant-equivalent characteristics that justify its inclusion in the high-risk group for perioperative stratification: (1) locally aggressive growth with intravascular extension and potential for distant metastasis via venous channels; (2) life-threatening complications including cardiac outflow obstruction, heart failure, and sudden death, with reported mortality in 10-30% of advanced cases (3–5); (3) surgical complexity requiring extensive resection, vascular reconstruction, and frequently cardiopulmonary bypass, technical demands comparable to malignant retroperitoneal sarcomas (6); and (4) high recurrence rate (13-30%) necessitating long-term surveillance similar to malignant tumors (5). Recent single-center report by Wen et al. declared that perioperative risk assessment should prioritize biological behavior and clinical risk over pure histopathological diagnosis (11). Therefore, we maintain that our classification strategy is clinically appropriate, methodologically sound, and consistent with current literature on IVL management (12).

Patients with malignant tumors were significantly older than those with non-malignant tumors (57.17 ± 13.41 years vs. 41.11 ± 19.80 years), which may reflect the cumulative, multistep processes involved in malignant tumor development (13). In contrast, the growth of non-malignant giant abdominal masses appears to be more strongly influenced by genetic and hormonal factors and demonstrates a weaker association with age. Surgical duration was significantly longer in the malignant tumor group than in the non-malignant tumor group, with medians of 260 (175, 308) min and 158 (150, 180) min, respectively (*p* < 0.05), indicating greater surgical complexity and a higher likelihood of extensive resection, lymph node dissection, or combined organ resection (14, 15).

### 4.2 Value of optimized preoperative preparation

In this cohort, 52.4% of patients underwent multidisciplinary team (MDT) consultation, 57.1% received preoperative tumor vascular embolization, and 33.3% underwent double-J ureteral stent placement, reflecting comprehensive preparation for complex surgical procedures. Stratified analysis indicated that patients with giant retroperitoneal or abdominopelvic masses associated with severe compression of adjacent organs had significantly higher rates of MDT consultation than those with giant abdominal wall masses (*p* < 0.05), underscoring the increased clinical attention required for high-risk masses (16).

Preoperative interventional embolization was performed in 57.1% of patients. The primary objectives of embolization include reduction of tumor blood supply, decrease in tumor volume, and protection of critical anatomical structures (17). In this study, massive hemorrhage (≥ 1,000 mL) occurred in 57.1% of patients. Although this proportion was relatively high, it remains acceptable given the complexity of giant abdominal masses and the fact that not all patients underwent preoperative arterial embolization. Median blood loss and transfusion volume in the retroperitoneal mass group reached 3,000 mL and 4,100 mL, respectively, highlighting the particular importance of preoperative embolization in cases with anticipated substantial intraoperative bleeding.

Double-J ureteral stent placement was performed in 33.3% of patients, primarily to protect the ureters. Stratified analysis demonstrated significantly higher utilization in the abdominopelvic mass group than in other anatomical groups (*p* < 0.05), reflecting recognition of the increased risk of ureteral injury in these patients. Giant abdominopelvic masses may compress the ureters or cause hydronephrosis preoperatively, and ureteral injury represents a major surgical risk. Preoperative stent placement can relieve hydronephrosis, facilitate intraoperative identification of the ureters, and provide structural support in the event of injury, thereby reducing postoperative complications (18).

### 4.3 Stratified management of anesthesia and monitoring

In this study, 76.19% of patients received general anesthesia with endotracheal intubation, whereas 19.05% received general anesthesia with a laryngeal mask airway. This distribution reflects an individualized approach to airway management based on surgical complexity and patient condition. One patient undergoing resection of a giant abdominal wall mass required partial rib resection because of chest wall involvement; therefore, double-lumen endotracheal intubation was selected to permit one-lung ventilation if necessary. No patients received combined neuraxial anesthesia, which may be attributable to the complex tumor location, extensive surgical fields, significant circulatory effects, and difficulties related to positioning for neuraxial puncture (1). Nevertheless, combined neuraxial anesthesia may remain an option in carefully selected patients with localized abdominal wall masses and favorable general conditions.

Bilateral transversus abdominis plane block was applied as adjunctive analgesia in 28.57% of patients. This technique is associated with reduced requirements for general anesthetic agents, improved postoperative analgesia, and facilitation of enhanced recovery after surgery pathways (19). Broader application of this technique may be beneficial, particularly in patients with abdominal wall masses.

Invasive arterial pressure monitoring was used in 76.2% of patients, central venous pressure monitoring in 71.4%, and FloTrac-based cardiac output monitoring in 38.0%. This monitoring strategy reflects the emphasis on hemodynamic surveillance during high-risk procedures. Invasive arterial pressure monitoring enables continuous real-time assessment, facilitates arterial blood gas analysis, and guides vasoactive drug administration. Central venous pressure monitoring assists with volume assessment and vasoactive agent infusion. FloTrac monitoring provides advanced hemodynamic parameters, including cardiac output, stroke volume, and systemic vascular resistance, and is particularly valuable for circulatory management in patients with retroperitoneal masses who are prone to pronounced hemodynamic instability (16).

Invasive arterial pressure monitoring was used in 76.2% of patients, central venous pressure monitoring in 71.4%, and FloTrac-based cardiac output monitoring in 38.0%. This monitoring strategy reflects contemporary emphasis on hemodynamic surveillance during high-risk procedures. Recent consensus recommendations for major abdominal surgery advocate goal-directed hemodynamic therapy (GDHT) utilizing dynamic preload indices (SVV ≤10–12%) coupled with cardiac index targets (CI ≥2.5 L min^-1^ m^-2^) to individualize fluid and vasoactive drug administration (20, 21). A 2024 randomized trial demonstrated that GDFT guided by pulse-contour analysis or esophageal Doppler reduced intraoperative blood loss by 37% and accelerated postoperative recovery compared with conventional management (22). While our monitoring approach aligns with these principles, future implementation of standardized GDHT algorithms with predefined hemodynamic targets may further optimize outcomes in giant abdominal mass resection.

### 4.4 Management characteristics and risk stratification by anatomical location

Retroperitoneal masses posed the greatest challenges. Median blood loss in the retroperitoneal group was 3,000 mL, compared with 400 mL in the abdominal wall group and 1150 mL in the intra-abdominal group. Although these differences did not reach statistical significance, the observed trend remains clinically relevant. Median transfusion volume was also highest in the retroperitoneal group at 4100 mL. ICU stay was significantly longer in this group, with a median of 5 days (*p* < 0.05), reflecting greater disease severity and surgical risk. Contributing factors include the deep anatomical location, rich vascular supply, pronounced circulatory effects, and extensive surgical fields. Key anesthetic management considerations include comprehensive preoperative assessment, enhanced intraoperative hemodynamic monitoring, meticulous volume management, and proactive vasoactive drug administration.

For intra-abdominal masses, median blood loss of 1,150 mL was lower than that observed in the retroperitoneal group but remained clinically significant. Compression of major vessels, such as the inferior vena cava and portal vein, can impair venous return and portal blood flow, resulting in relative hypovolemia and portal hypertension. Diaphragmatic compression may also compromise pulmonary function. During anesthesia induction, appropriate positioning is essential to reduce the risk of regurgitation, aspiration, and hypoxemia, and awake tracheal intubation may be considered when indicated (23). During anesthesia maintenance, enhanced respiratory monitoring and optimized ventilation strategies are required to reduce the risk of postoperative pulmonary complications, including atelectasis.

Abdominal wall masses offer relative management advantages because of their superficial location and less pronounced effects on respiratory and circulatory function. Supraglottic airway devices or regional anesthesia techniques may be considered in selected cases. Management priorities include optimization of patient positioning and the use of regional anesthesia techniques or general anesthesia combined with regional blocks for perioperative analgesia. Large abdominal wall defects following mass resection also warrant attention, as extensive defects increase the risk of postoperative fluid collection and surgical site infection (24).

It should be noted that although the retroperitoneal tumor group showed a trend of greater blood loss and transfusion volume, and the effect size suggested moderate to large clinical differences, these findings did not reach the traditional statistical significance level due to sample size limitations. Therefore, the current conclusion should be regarded as an exploratory discovery that needs to be further validated through multi center large sample studies.

### 4.5 Key strategies for fluid management and complication prevention

Median crystalloid and colloid infusion volumes were 3,500 mL and 500 mL, respectively, reflecting the application of goal-directed fluid therapy principles. Fluid management during resection of giant abdominal masses is particularly challenging because of difficulties in accurate volume assessment, high bleeding risk, and marked circulatory fluctuations. Blood transfusion was required in 57.1% of patients, and vasoactive agents were administered in 66.7%, with norepinephrine being the most frequently used agent (61.9%). These findings underscore the substantial hemorrhagic risk associated with these procedures and the need for vigilant circulatory support.

The overall complication rate was 52.6%, with major complications including surgical site infection, bilateral lower-lobe atelectasis, and hemorrhagic shock. No perioperative mortality occurred. Although the complication rate was relatively high, it remains acceptable given the technical complexity of these procedures, and all complications were managed effectively without resulting in severe adverse outcomes. Preventive strategies should emphasize strict aseptic technique, meticulous hemostasis, enhanced postoperative monitoring, multidisciplinary collaboration, and appropriate prophylactic interventions (25).

### 4.6 Clinical recommendations and standardized protocols

#### 4.6.1 Standardization of preoperative assessment

A standardized preoperative assessment protocol is essential and should include systematic evaluation of ASA classification, tumor location, tumor size, and comorbidities. Individualized anesthetic plans should be developed based on tumor characteristics and patient comorbidities, with monitoring strategies tailored to anatomical location and surgical risk.

#### 4.6.2 Individualized strategies

For abdominal wall masses, management should prioritize optimization of patient positioning and respiratory monitoring, with consideration of general anesthesia combined with regional blocks. For intra-abdominal masses, emphasis should be placed on volume management and preservation of diaphragmatic function, supported by comprehensive hemodynamic monitoring. For retroperitoneal masses, particular attention should be directed toward maintaining circulatory stability through intensive monitoring and the judicious use of vasoactive agents (26).

#### 4.6.3 Multidisciplinary collaboration

Preoperative MDT consultation involving anesthesiology, surgery, radiology, and pathology is essential for the development of optimal management strategies. Close intraoperative collaboration supports procedural safety, and coordinated postoperative management facilitates recovery and reduces the risk of complications (27, 28).

The application of Enhanced Recovery After Surgery (ERAS) principles in complex retroperitoneal procedures represents an important direction for future practice. A 2024 multi-center study developed an ERAS protocol specifically for pediatric patients undergoing abdominal and retroperitoneal tumor resections, demonstrating feasibility and potential benefits in reducing complications and expediting recovery (29). Integration of goal-directed fluid therapy within ERAS pathways is increasingly recognized as essential for high-risk abdominal surgery (30, 31). Recent evidence suggests that GDFT utilizing dynamic parameters (SVV, PPV) to guide fluid administration reduces length of stay and complications, while avoiding both hypovolemia (risk of acute kidney injury) and hypervolemia (risk of delayed bowel function) (30, 32). Systematic adoption of comprehensive ERAS protocols, incorporating prehabilitation, multimodal analgesia, and standardized hemodynamic management, may further enhance perioperative outcomes in adult patients undergoing giant abdominal mass resection.

### 4.7 Limitations and future perspectives

#### 4.7.1 Limitations

Several limitations should be acknowledged. The retrospective design may have been affected by incomplete data documentation, and selection bias may limit the generalizability of the findings. The small sample size reduced statistical power and may have limited the ability to detect true differences between groups. In addition, the limited sample size precluded multivariable analysis, thereby preventing the identification of independent risk factors. As a case series, the level of evidence remains relatively low, highlighting the need for studies with stronger methodological designs.

#### 4.7.2 Future research directions

Future investigations should focus on prospective multicenter studies to increase sample size and enhance external validity. Further research is also warranted to evaluate the impact of different intraoperative monitoring strategies on patient outcomes, to develop risk prediction models to support clinical decision-making, and to promote the systematic integration of enhanced recovery after surgery principles into perioperative management for resection of giant abdominal masses.

## 5 Conclusions

Anesthetic management for resection of giant abdominal masses requires individualized planning and stratified strategies based on anatomical location and pathological characteristics. Preoperative multidisciplinary team consultation and interventional embolization contribute to perioperative risk reduction. During surgery, particular emphasis should be placed on advanced hemodynamic monitoring and meticulous volume management, especially in patients with retroperitoneal masses, in whom intensified circulatory surveillance is essential. The application of multimodal analgesia and enhanced recovery after surgery principles may further improve perioperative outcomes.

In summary, this study provides clinically relevant insights into anesthetic management for resection of giant abdominal masses and offers a preliminary framework for standardized perioperative management of these rare and complex cases. With continued accumulation of clinical experience and advances in perioperative technology, further improvements in safety and effectiveness can be anticipated.

## Data Availability

The original contributions presented in the study are included in the article/supplementary material. Further inquiries can be directed to the corresponding author.
